# Strategies for Developing Sensitive and Automated LC-MS/MS Assays of a Pharmaceutical Compound and Its Metabolite from Whole Blood Matrix

**DOI:** 10.3390/pharmaceutics2020159

**Published:** 2010-04-30

**Authors:** Raymond N. Xu, Jill Polzin, Michelle Kranz, Phillip Vaca, Maria Metchkarova, Matthew J. Rieser, Tawakol A. El-Shourbagy

**Affiliations:** Department of Drug Analysis, Abbott Laboratories, 100 Abbott Park Road, Abbott Park, IL 60064, USA; E-Mails: Jill.Polzin@abbott.com (J.P.); michelle.kranz@abbott.com (M.K.); phillip.vaca@abbott.com (P.V.); maria.metchkarova@abbott.com (M.M.); matthew.j.rieser@abbott.com (M.J.R.); Tawakol.El-Shourbagy@abbott.com (T.A.E.-S.)

**Keywords:** high throughput analysis, whole blood matrix, liquid chromatography, tandem mass spectrometry, automation, prodrug, incurred sample reproducibility, metabolite, extraction

## Abstract

When compared with biological samples in other matrices (plasma, urine, *etc*.) that are typically seen in bioanalytical applications, whole blood samples present unique challenges in method development, because of the viscous nature of blood and complexity of its constituents. In this article, we have developed and validated a series of quantitative bioanalytical methods for the determination of a pharmaceutical compound, Compound A, and its phosphate metabolite from whole blood matrices using liquid chromatography tandem mass spectrometry. All methods employed a simple protein precipitation procedure that was automated in 96-well format. The methods were subjected to vigorous tests in precision, accuracy, matrix effect, reproducibility, and robustness. Monolithic chromatography was used to improve sample throughput in one of the methods. The results also demonstrated that proper sample preparation procedures, such as sample transfer and lysing of blood cells prior to the extraction, are key to reproducible results for pharmacokinetic parameter determination.

## 1. Introduction

In pharmaceutical discovery and development, biological fluids such as plasma, serum, whole blood, and urine are most commonly analyzed for pharmacokinetic parameter evaluation. Most bioanalytical applications are from plasma samples. But for many compounds such as cyclosporin A (CsA) that mainly distributes in the erythrocyte, whole blood rather than plasma or serum is the matrix of choice for the measurement of drug exposure in animal or human subjects. Whole blood samples present unique challenges in method development and validation because of the viscous nature of blood and complexity of its constituents. Of all the current techniques available, LC-MS/MS has been generally accepted as the preferred technique for quantitative and analysis of small-molecule drugs, metabolites, and other xenobiotic molecules in biological matrices including liquid whole blood samples due to its inherent specificity and sensitivity [1,2].

Sample preparation techniques for whole blood assays are as diverse as those for plasma assays [3,4,5,6,7,8,9,10,11]. Although simple sample preparation techniques like protein precipitation (PPT) have been used in the whole blood analysis, whole blood analysis typically calls for more labor-intensive treatment such as liquid-liquid extraction (LLE) or solid-phase extraction (SPE). In some cases, PPT is used as a pretreatment method prior to LLE or SPE. Among them, a method for the determination of indapamide in human whole blood has been developed with a sensitivity of 0.5 ng/mL as the lower limit of quantification (LLOQ). The procedure for the extraction of indapamide and glimepiride as internal standard (IS) involves hemolysis and deproteination of whole blood using zinc sulfate (ZnSO_4_) followed by liquid–liquid extraction using ethyl acetate [3]. The sample extracts after drying are reconstituted and analyzed by LC–MS/MS. The mean recovery for indapamide was 82.40% and 93.23% for IS. The total run time was 2.5 min to monitor both indapamide and the IS. The method was validated over the range of 0.5–80.0 ng/mL and applied to sample analysis of a bioequivalence study for 1.5 mg sustained-release formulations. A specific LC-MS/MS method was reported for the determination in human whole blood of Aplidin, a novel depsipeptide under investigation in clinical studies [4]. Didemnin B was used as internal standard. After protein precipitation with acetonitrile and liquid–liquid extraction with chloroform, APL was separated by liquid chromatography using a gradient program. A combination with PPT with SPE was used as the extraction approach in the simultaneous determination of six beta-blockers and three calcium-channel antagonists from human whole blood [5]. Sample clean-up was achieved by solid phase extraction (SPE) with a mixed-mode column after protein precipitation. 

While most of whole blood methods reported to date employed manual extraction, 96-well techniques have been developed for whole blood analysis. Ji *et al.* reported a quantitative method for the analysis of ABT-578 in human whole blood samples. Sample preparation was achieved by a semi-automated 96-well format LLE method [6]. Aluminum/polypropylene heat seal foil was used to enclose each well of the 96-well plate for the liquid-liquid extraction. An LC-MS/MS method with pre-column regeneration was developed for the analysis of sample extracts. The ammonium adduct ions generated from electrospray ionization were monitored as the precursor ions. The assay was validated for a linear dynamic range of 0.20–200.75 ng/mL. A 96-well based LC-MS/MS method was developed and validated for the determination of N-methyl-4-isoleucine-cyclosporin (NIM811) over the concentration range of 1–2500 ng/mL in human whole blood using a 0.05 mL sample volume [7]. NIM811 and the internal standard, d12-cyclosporin A (d12-CsA) were extracted from blood using Methyl *tert*-butyl ether (MTBE) via liquid–liquid extraction in 96-well plate. After evaporation of the organic solvent and reconstitution, a 10 μL aliquot of the resulting extract was injected onto the LC-MS/MS system. The method was used to measure the exposure of NIM811 in human subjects.

In this article, we described our strategies in developing sensitive and automated LC-MS/MS assays from whole blood matrix for Compound A, a pharmaceutical compound that is currently under development by Abbott Laboratories. Compound A is a prodrug and has an active metabolite in phosphate form.

## 2. Experimental Section

### 2.1. Chemicals

Acetonitrile, methanol, and formic acid (FA) were purchased from EM Science (Gibbstown, NJ, USA). Ammonium acetate, in ACS grade, was purchased from J.T. Baker (Phillipsburg, NJ, USA). Water was produced by a Millipore (Bedford, MA, USA) Milli-Q unit. Compound A, its active metabolite, and their respectively d4-labled internal standards were obtained from Abbott Laboratories (Abbott Park, IL, USA). Normal rat, dog, and rabbit whole blood with potassium EDTA as anticoagulant was purchased from Biological Specialties Corporation (Colmar, PA, USA).

### 2.2. Standard and quality control (QC) solutions

Stock solution of Compound A was prepared in 50/50 (v/v) acetonitrile/water. Stocks for the active metabolite and the two internal standards were diluted in 80/20 (v/v) methanol/water. The stock solutions of the internal standard were further diluted with 30/70 (v/v) methanol/water for internal standard working solution preparation. The stock solutions for the calibration standards and quality control samples were prepared from separate measurements. The solutions were stored in plastic bottles in a refrigerator.

### 2.3. Sample preparation

All steps of sample preparation were handled in a semi-automated fashion. Sample transfer steps were performed by a liquid handler with positive displacement capability (Hamilton Lab AT 2 Plus, Reno, Nevada, USA). Samples are thawed completely in cold water while sonicating. Each sample is thoroughly mixed by vortexing prior to taking the aliquot.

For rat whole blood method, 60 μL of working internal standard solution in 30/70 (v/v) methanol/water was added to each appropriate well of a clean 2 mL square well 96-well plate to lyse the red blood cells. After 40 μL of rat whole blood sample was added to the appropriate wells, the plate was vortexed for approximately 10 minutes. Then 500 μL of 20/80 (v/v) methanol/acetonitrile solution was added to each well. The plate was vortexed and centrifuged to sediment precipitate. Supernatant (490 µL) was transferred to a clean 1 mL round well 96-well plate and dried completely under a stream of nitrogen. The extract was reconstituted with 180 μL of 20/80 (v/v) acetonitrile/water and 20 μL was injected to the LC-MS/MS.

The sample preparation procedure for the dog whole blood method and rabbit whole blood method was essentially the same as that for the rat whole blood method. The only difference was that dog whole blood method and rabbit whole blood method employed an injection volume of 30 µL.

### 2.4. Chromatography System for rat and dog whole blood method

An HPLC system was used to perform separation and to introduce sample into the detector. A Shimadzu LC-10ADvp pump (Shimadzu, Columbia, MD, USA) was used to deliver mobile phase, which consisted of 0.2% Formic Acid in 35/65 (v/v) acetonitrile/ water. The mobile phase, with a flow rate of 0.3 mL/min, was used to perform separation on the guard and analytical column (Thermo Hypersil Gold, 50 × 2.1 mm, 5 μm, Pittsburgh, PA). Both Phenomenex Security Guard C12 4 × 2.0 mm (Torrance, CA) and Thermo Hypersil Gold, 5 μm, 10 × 2.1 mm Drop in Guard (Pittsburgh, PA) have been validated as the guard column. A Shimadzu SIL-HT_C_ autosampler /controller was used to inject samples. A column-switching valve (Valco Instruments, Houston, TX, USA) was used to direct the sample to the analytical column through the guard column or bypassing it. Another similar switching valve was used to direct the flow from the system to either the mass spectrometer or to waste collection. An Agilent 1100 pump (Hewlett-Packard, Waldbronn, Germany) with a two-way solvent selector (Parker Instrumentation, Fairfield, NJ) was used to deliver solvents for guard column back wash. The guard column was switched offline at 1.1 min after the injection and backwashed with 95/5 (v/v) acetonitrile/water at a flow rate of 1.5 mL/min. The guard column was switched online at 3.1 min after the injection and equilibrated with the mobile phase. The run time was approximately 4.5 minutes.

### 2.5. Chromatography system for rabbit whole blood method

The Shimadzu LC-10ADvp pump (Shimadzu, Columbia, MD, USA) was used to deliver mobile phase, which consisted of 5 mM ammonium acetate and 0.5% formic acid in 35/65 (v/v) acetonitrile/water. The mobile phase with an isocratic flow rate of 0.8 mL/min was used to perform separation on the analytical column (Merck KGaA, Chromolith Fast Gradient RP-18e, 50 × 2 mm). A Merck KGaA, Chromolith Guard Cartridge (RP-18e, 10 × 4.6 mm) was used as the guard column. The Shimadzu SIL-HTC autosampler /controller was used to inject samples. A Valco switching valve was used to direct the flow from the system to either the mass spectrometer or to waste collection. The run time was approximately 3.0 min.

### 2.6. Mass Spectrometric detection

LC-MS/MS detection was performed using an API 4000 (Applied Biosystems, Toronto, ON, Canada) triple-quadrupole mass spectrometer with an electrospray ionization source operated in the positive ion mode. The computer control system was Analyst^TM^ version 1.4.2. The following transition channels were used in selected reaction monitoring (SRM) detection of the analytes: m/z 320.1 → 285.2 for Compound A, m/z 324.2 → 289.2 for d4-Compound A, m/z 400.3 → 169.2 for the metabolite, and 404.4 → 171.2 for d4-metabolite.

### 2.7. Calibration curves and quantitation of samples

Analyst^TM^ version 1.4.2 was used for the data acquisition, peak area integration, regression analysis, and quantitation. For each analytical batch, a calibration curve was derived from the peak area ratios (analyte/internal standard) using weighted linear least-squares regression of the area ratio *versus* the concentration of the standards. A weighting of 1/x^2^ (where x is the concentration of a given standard) was used for curve fitting. The regression equation for the calibration curve was used to back-calculate the measured concentration at each standard level and the results were compared with the theoretical concentration to obtain the accuracy, expressed as a percentage of the theoretical value, for each standard level measured.

## 3. Results and Discussion

### 3.1. Strategies used in method development

Both Compound A and its active metabolite were analyzed with positive ion electrospray mode. The metabolite, a phosphate, has the best sensitivity when ionized in negative mode. However, the positive mode offered adequate sensitivity to achieve the lower limit of quantitation demonstrated in this article. 

During method development, various extraction approaches were experimented for optimum recovery and automation feasibility. Compound A can be easily extracted from whole blood by organic solvent such as ethyl acetate or methyl tert-butyl ether, however, the metabolite has poor recovery when liquid-liquid extraction is used. This was possibly due to the zwitterions-like property of the metabolite. Off-line solid phase extraction has been attempted, but the procedure was deemed too labor intensive for routine operation to support the development program. Simple protein precipitation (PPT) procedures offered good recovery (95% to 100%) for both compounds from the whole blood matrix. PPT approach was fully evaluated later for method robustness because of the fact that extract from PPT is usually less clean than others. A guard column back wash procedure was implemented for rat and dog whole blood methods so that the guard cartridge was cleaned after each injection and reconditioned for the next injection. Both rat and dog methods for whole blood samples were validated in such fashion.

One of the major advantages of PPT is that it can be easily automated in 96-well format. Whole blood samples are typically more difficult to work with than plasma samples because of their viscous nature. When using robotic liquid handler to pipette whole blood samples, it was found that the aspiration speed of the liquid handler has to be adjusted properly to ensure accurate aliquoting of whole blood sample. Generally, aspiration speed of the liquid handler for the whole blood samples needs to be considerably slower than that for plasma samples.

The same sample preparation approach was used for rabbit whole blood method. To improve the throughput of the assay, monolithic chromatography was investigated for the separation of the analytes. The monolithic column was operated at a flow rate of 0.8 mL/min, which was much higher than 0.3 mL/min flow rate used in rat and dog whole blood methods. But the back-pressure on the monolithic column remained low at approximately 50 bar and the separation of Compound A and metabolite was satisfactory.

### 3.2. Rat and dog whole blood method performance

Precision and accuracy of each method was validated by three consecutive analytical batches. Each batch contained a single set of calibration standards, six replicates of QCs at three concentration levels, six replicates of LLOQ (lower limit of quantitation) evaluation samples, and six replicates of ULOQ (upper limit of quantitation) evaluation samples. Each batch also contained other test samples such as system suitability sample. 

Statistical data for LLOQ, ULOQ, and QC samples for rat whole blood method are summarized in Table 1. The data show that this method is consistent and reliable with low %CV and %bias values. The accuracy (%bias) at the lower limit of quantitation (LLOQ) for Compound A was 2.3% and the precision (%CV) at the LLOQ was 3.4%, while the accuracy at LLOQ for the metabolite was 10.2% and the precision at LLOQ was 10.9%. The inter-day %bias and %CV of all quality control samples including ULOQ of Compound A were within ±5.5% and ≤2.3%, respectively. The inter-day %bias and %CV of all quality control samples including ULOQ of the metabolite were within ±11.1% and ≤4.9%, respectively. 

**Table 1:** Inter-day accuracy and precision of the LLOQ, low QC (LQC), mid QC (MQC), high QC (HQC), and ULOQ evaluation samples for rat whole blood method. Mean values in the table are the average of the back-calculated concentrations from the standard curve.

QC sample (ng/mL)	Compound A					Metabolite				
	LLOQ	LQC	MQC	HQC	ULOQ	LLOQ	LQC	MQC	HQC	ULOQ
	4.36	11.0	137	1,720	2,180	4.59	11.7	147	1,830	2,300
n	18	18	18	18	18	18	18	18	18	18
Mean	4.46	11.6	144	1,760	2,160	5.06	13.0	159	1,930	2,300
CV (%)	3.4	2.2	2.3	2.3	2.6	10.9	4.9	2.3	1.9	2.0
Bias (%)	2.3	5.5	5.1	2.3	-0.9	10.2	11.1	8.2	5.5	0.0

**Figure 1:**
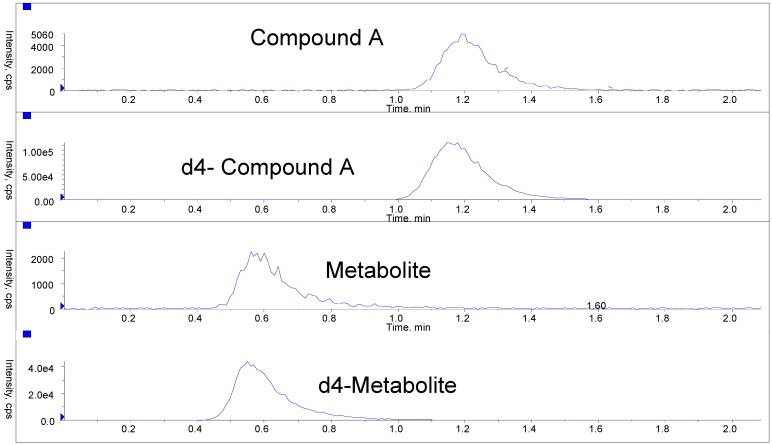
Representative ion chromatograms of an LLOQ sample from the rat whole blood method.

Representative ion chromatograms of an LLOQ sample are shown in Figure 1. To evaluate extraction recovery, three levels of recovery control solutions in neat solution were prepared at the same concentrations as those of whole blood quality control samples. The peak areas of extracted samples were compared to the average peak areas of the control samples at each concentration level. An extraction recovery of 56% and 42% was determined for Compound A and the metabolite, respectively. To evaluate matrix effect of the method, low QC samples were prepared using six different lots of rat blood that was not used to prepare standards and regular QCs. The percent difference was computed using the mean of the calculated concentrations and theoretical concentrations. The mean difference was found to be between -5.5% and 0.0% for each lot tested for Compound A, and between -6.2% and -2.7% for each lot tested for the metabolite.

Dog whole blood method had similar performance to that of the rat whole blood method. Statistical data for LLOQ, ULOQ, and QC samples for dog whole blood method are summarized in Table 2. The accuracy (%bias) at the lower limit of quantitation (LLOQ) for Compound A was 6.5% and the precision (%CV) at the LLOQ was 4.7%, while the accuracy at LLOQ for the metabolite was 6.0% and the precision at LLOQ was 14.5%. The inter-day %bias and %CV of all quality control samples including ULOQ of Compound A were within ±5.1% and ≤5.0%, respectively. The inter-day %bias and %CV of all quality control samples including ULOQ of the metabolite were within ±3.9% and ≤8.0%, respectively. 

**Table 2:** Inter-day accuracy and precision of the LLOQ, LQC, MQC, HQC, and ULOQ evaluation samples for the dog whole blood method. Mean values in the table are the average of the back-calculated concentrations from the standard curve.

QC sample (ng/mL)	Compound A					Metabolite				
	LLOQ	LQC	MQC	HQC	ULOQ	LLOQ	LQC	MQC	HQC	ULOQ
	10.8	22.1	276	2,300	2,730	3.17	6.72	84.0	700	811
n	18	18	18	18	18	18	18	18	18	18
Mean	11.5	22.9	290	2,350	2,680	3.36	6.98	84.9	721	835
CV (%)	4.7	5.0	2.5	4.8	5.0	14.5	8.0	3.3	4.4	4.0
Bias (%)	6.5	3.6	5.1	2.2	-1.8	6.0	3.9	1.1	3.0	3.0

**Figure 2:**
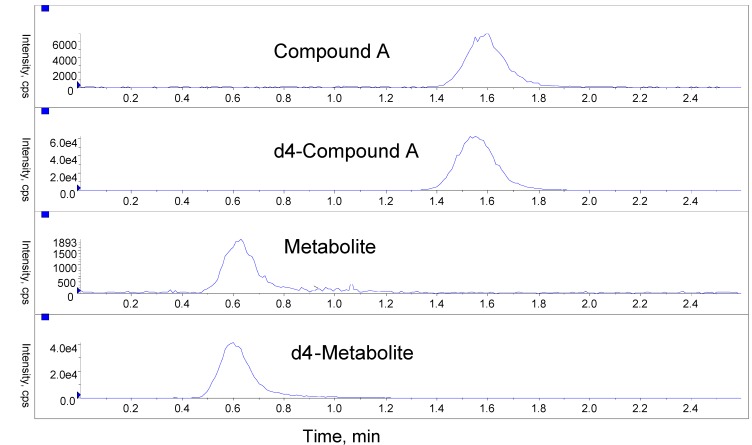
Representative ion chromatograms of an LLOQ sample from the dog whole blood method.

Representative ion chromatograms of an LLOQ sample are shown in Figure 2. An extraction recovery of 41% and 46% was determined for Compound A and the metabolite, respectively. Low QC (LQC) sample prepared from six different lots of dog blood were used to quantitatively measure matrix effect of the dog whole method. Mean difference was found to be between -14.8% and 1.4% for each lot tested for Compound A, and between -9.0% and -2.7% for each lot tested for the metabolite.

### 3.3. Reproducibility testing

Reproducibility testing has profound impact on bioanalytical method development and validation processes. Recently, such testing has been implemented as mandatory repeat experiment using incurred samples for regulated bioanalytical studies in pharmaceutical industry. The results from mandatory repeat experiment are often treated as a part of method validation. Currently, there are very limited publications on incurred sample reproducibility (ISR) for whole blood analytical methods. 

Both rat and dog whole blood methods were initially developed with working internal standard solution prepared in 80/20 (v/v) methanol/water. The working internal standard solution was used to lyse blood cells by a brief mixing step. The sample preparation procedures with this solution worked very well for the spiked sample, *i.e.*, standards and QCs. The procedures were used for sample analysis of two GLP toxicology studies. Incurred sample repeat were performed for both studies. The acceptance criterion was that the repeat value must be within ± 20% of the original result for two-thirds of the repeated samples. One of the two studies was a rat four-week toxicity study. The measured difference for Compound A was between -9.1% and 40.9% for 24 repeat samples and 19 out of 24 of the repeat sample results were within ±20% of the original. The measured difference for the metabolite was between -1.9% and 12.1% for all 24 repeat samples. However, incurred sample repeat test failed for the other study, which was a dog four-week toxicity study. The results showed that 10 out of 24 repeat samples had %bias greater than ±20% for Compound A and 13 out of 24 repeat samples had %bias greater than ±20% for the metabolite. 

An in-study investigation was conducted to understand why the method performed well for spiked samples, but not for the incurred samples. It is suspected that inhomogeneity of the dog blood samples caused the failure of the incurred sample repeat. A multi-tube vortexer has been used to mix thawed study samples and then samples were transferred by robotic liquid handler to a 96-well plate. It was hypothesized that some of the blood samples may not be homogeneous after mixing on a multi-tube vortexer because of the highly viscous nature of the whole blood and relatively large volume (approximately 1 mL each) of the dog blood samples. An action was taken to manually pipette samples to the 96-well plate and ensure that each sample was thoroughly mixed by vortexing prior to taking the aliquot. Despite some improvement made by improving sample homogeneity in this way, the retest results still failed the acceptance criteria.

It was later realized that the high organic content used in the lysing solution, *i.e.*, the working internal standard solution prepared in 80/20 (v/v) methanol/water, may have caused unexpected protein precipitation before the blood cells were fully lysed. A new working internal standard solution was prepared in 30/70 (v/v) methanol/water as the lysing solution and the lysing time was well defined to be a 10 min vortexing after the lysing solution was added. The samples were still added manually to the 96-well plate. The changes to working internal standard solution was first qualified using spiked calibration standard and quality control samples and then applied to the analysis of study samples, which were reassayed later as incurred sample repeats. The implement of the changes led to all 48 incurred sample repeats meeting the acceptance criteria for both Compound A and the phosphorylated metabolite. The measured difference for Compound A was between -15.5% and 7.6% and measured difference for the metabolite was between -11.4% and 5.6%. The same 48 incurred samples were reanalyzed later with the same revised procedures, but with samples pipetted by robotic liquid handler. Again, all 48 samples met acceptance criteria for both analytes. Such procedural changes were also later implemented to the rat whole blood method. 

### 3.4. The use of monolithic chromatography in rabbit whole blood method

**Figure 3:**
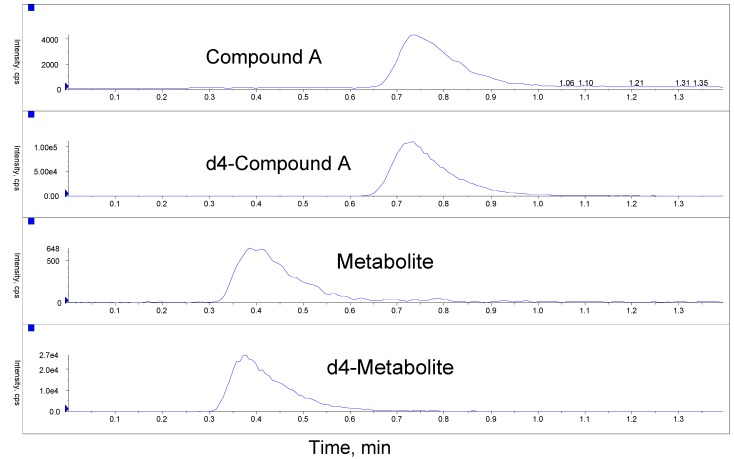
Representative ion chromatograms of an LLOQ sample from the rabbit whole blood method.

The rabbit whole blood method was later validated by using monolithic chromatography [12,13,14], which significantly reduced run time from that in rat and dog whole methods. Guard column backwash and regeneration approach was not implemented in rabbit whole blood method. However, to our surprise, the method worked well with isocratic separation on the Chromolith guard cartridge and Chromolith analytical column. Statistical data for LLOQ, ULOQ, and QC samples for the rabbit whole blood method are summarized in Table 3. The accuracy (%bias) at the lower limit of quantitation (LLOQ) for Compound A was 6.2% and the precision (%CV) at the LLOQ was 4.2%, while the accuracy at LLOQ for the metabolite was 3.7% and the precision at LLOQ was 6.3%. The inter-day %bias and %CV of all quality control samples including ULOQ of Compound A were within ±5.0% and ≤3.5%, respectively. The inter-day %bias and %CV of all quality control samples including ULOQ of the metabolite were within ±3.8% and ≤4.8%, respectively. Representative ion chromatograms of an LLOQ sample are shown in Figure 3. 

**Table 3:** Inter-day accuracy and precision of the LLOQ, LQC, MQC, HQC, and ULOQ evaluation samples for the rabbit whole blood method. Mean values in the table are average of the back-calculated concentrations from the standard curve.

QC sample (ng/mL)	Compound A					Metabolite				
	LLOQ	LQC	MQC	HQC	ULOQ	LLOQ	LQC	MQC	HQC	ULOQ
	4.05	10.5	131	1,640	2,030	4.04	10.5	132	1,650	2,020
n	18	18	18	18	18	18	18	18	18	18
Mean	4.30	10.5	127	1,630	2,080	4.19	10.9	130	1,620	1,960
CV (%)	4.2	2.5	3.4	3.5	2.7	6.3	4.8	2.3	2.0	1.8
Bias (%)	6.2	0.0	5.0	-0.6	2.5	3.7	3.8	-1.5	-1.8	-3.0

An extraction recovery of 45% and 44% was determined for Compound A and the metabolite, respectively. Low QC sample prepared from six different lots of rabbit blood were also used to quantitatively measure matrix effect of the dog whole method. Mean difference was found to be between -11.7% and 1.1% for each lot tested for Compound A, and between -1.9% and 10.9% for each lot tested for the metabolite. The rabbit whole blood method was later applied to a reproductive toxicity study and yielded satisfactory assay performance.

## 4. Conclusions

In conclusion, we have developed bioanalytical methods for quantitative determination of a pharmaceutical compound and its phosphorylated metabolite from whole blood matrix to meet preclinical development need of the drug candidate. A simple sample preparation method, *i.e.*, protein precipitation method, can be used for whole blood sample analysis in 96-well format and was automated in simultaneous determination of the parent compound and the metabolite from whole blood matrix. Our results demonstrate the importance of incurred sample repeats in whole blood sample analysis. Proper sample preparation procedures such as sample transfer and lysing of blood cells prior to the extraction are keys to reproducible results.
